# Impact of dietary Biocide clay on growth, physiological status, and histological indicators of the liver and digestive tract in Nile tilapia (*Oreochromis niloticus*)

**DOI:** 10.1038/s41598-025-89042-9

**Published:** 2025-02-13

**Authors:** Asmaa S. Abd El-Naby, Amel M. El Asely, Mona N. Hussein, Abd El-Rahman A. Khattaby, Haitham G. Abo-Al-Ela

**Affiliations:** 1 Fish Nutrition Department, Central Laboratory for Aquaculture Research, Agriculture Research Center, Abassa, Abu Hammad, Sharkia Egypt; 2 Department of Aquatic Animals Diseases and Management, Faculty of Veterinary Medicine, Benha University, Benha, 13736 Egypt; 3 Department of Histology, Faculty of Veterinary Medicine, Benha University, Benha, 13736 Egypt; 4 Department of production and Aquaculture systems, Central Laboratory for Aquaculture Research Centre, Agriculture Research Centre, Abassa, Abu Hammad, Sharkia Egypt; 5 Genetics and Biotechnology, Department of Aquaculture, Faculty of Fish Resources, Suez University, Suez, 43221 Egypt

**Keywords:** Feed additives, Growth performance, Gut health, Organic acids, Natural clay, Nile tilapia, Animal physiology, Marine biology

## Abstract

This study evaluated the effects of Biocide, containing silicon tetrahedrons and organic acids, on growth performance, feed utilization, immune response, and oxidative status in Nile tilapia (*Oreochromis niloticus*). A total of 300 Nile tilapia fingerlings (initial weight: 3.55 ± 0.01 g) were distributed across 15 tanks and fed diets containing 0.0 (control), 0.25, 0.5, 1, and 2 g kg⁻¹ Biocide for 90 days, with three replicate tanks per treatment. Biocide is enriched with organic acids (fumaric acid and citric acid) and amino acids (glutamine, tyrosine, methionine, serine, and threonine). Fish fed Biocide-supplemented diets demonstrated significantly improved growth performance, with the highest weight gain, feed conversion ratio, and protein efficiency ratio observed in the 1 g kg⁻¹ group. Survival rates did not differ significantly among treatments. Whole-body crude protein content peaked in the 1 g kg⁻¹ group, while moisture, lipid, and ash contents remained unchanged. Hematological parameters, including red blood cell count, hemoglobin concentration, and packed cell volume, improved significantly. Serum lipid profiles showed reduced cholesterol, triglycerides, low-density lipoprotein, and very-low-density lipoprotein levels, alongside increased high-density lipoprotein levels, particularly in the 1 g kg⁻¹ group. Antioxidant enzyme activities (catalase, glutathione peroxidase) and total antioxidant capacity were significantly elevated in the liver and intestine, while malondialdehyde levels decreased. Digestive enzyme activities (amylase, lipase, and protease) were markedly enhanced. Histopathological analysis revealed improved liver, stomach, and intestinal morphology, including increased mucous secretion and enhanced intestinal villi structure, in fish fed 1 g kg⁻¹ Biocide. In conclusion, Biocide supplementation, particularly at 1 g kg⁻¹, significantly improved growth performance, feed utilization, immune function, and antioxidant capacity in Nile tilapia. Notably, the findings highlight Biocide’s primary mode of action on gut health, underscoring its potential as a dietary additive for improving aquaculture productivity.

**Supplementary Information:**

The online version contains supplementary material available at 10.1038/s41598-025-89042-9.

## Introduction

Nile tilapia (*Oreochromis niloticus*) is one of the most widely cultivated fish species in aquaculture due to its adaptability to various environmental conditions, significantly contributing to global production increases^1^. As aquaculture intensifies, wild capture fisheries have experienced a corresponding decline, while the production of farmed food fish continues to rise^2^. In 2018, aquaculture accounted for 82 million metric tons of food fish production^3^.

Current aquaculture practices are increasingly shaped by global trends such as the demand for antibiotic-free production and the need for sustainability, both of which are integral to long-term profitability^4,5^. Sustainable aquaculture requires a balanced approach that ensures economic viability, ecological responsibility, and social equity^6,7^. One of the critical factors affecting sustainable production is nutrient absorption, as it directly influences both fish growth and health^8^. Therefore, proper feed formulations are necessary to meet nutrient requirements and prevent deficiencies that could negatively impact fish performance^8^. Optimizing fish health is vital for ensuring high productivity, prompting nutritionists to continually investigate strategies that enhance growth and immune function^9,10^.

A growing body of research is focused on the use of natural feed additives to promote health and growth in aquaculture. These include various compounds such as synbiotics, prebiotics, herbal extracts, organic acids, clays, and nucleotides, all of which have demonstrated potential benefits for enhancing fish performance^11–19^. Among these, clay minerals have gained attention due to their environmentally friendly, non-toxic properties and low cost^20^.

Several studies have highlighted the efficacy of clays in improving fish growth and health. For example, bentonite, a type of clay mineral, has been shown to adsorb harmful algal toxins and aflatoxins, thereby improving the growth and overall condition of Nile tilapia^21,22^. Similarly, sericite, a mica-based natural clay, has been found to enhance digestive enzyme activity, gut microbiota, blood profiles, and immune function in tilapia^23^. Additionally, fish fed natural clay zeolite have demonstrated improved protein efficiency ratio (PER), feed conversion ratio (FCR), weight gain, and lower serum levels of liver enzymes (e.g., alkaline phosphatase (ALP), aspartate aminotransferase (AST), and alanine aminotransferase (ALT)), indicating better overall health^24^.

Furthermore, clays such as exfoliated micronized montmorillonite, when combined with algae, have been shown to enhance feed utilization, gut health, immune responses, and growth performance in olive flounder (*Paralichthys olivaceus*), even when compared to diets with higher fish meal content^25^. Long-term studies on the use of montmorillonite in rainbow trout (*Oncorhynchus mykiss*) have also confirmed its safety and effectiveness in improving growth, immune function, and disease resistance^26^. The European Food Safety Authority (EFSA) has recommended montmorillonite-illite mixed-layer clay as a safe and effective technological feed additive for all animal species EFSA Panel^27^, . However, further studies are needed to elucidate the physiological responses associated with these compounds.

Soil can serve as a source of organic acids and various other compounds that naturally form through interactions among different substances within ecosystems^28,29^. Organic acids are potent modulators that promote growth, enhance digestive enzyme activities (e.g., lipase, amylase, and trypsin), and improve antioxidant capacities (e.g., superoxide dismutase [SOD] and malonaldehyde [MDA]) as well as non-specific immunity (e.g., alkaline phosphatase and lysozyme activities) in juvenile fish^30,31^. These acids have been shown to increase the total counts of erythrocytes, platelets, and leukocytes, along with hemoglobin content, hematocrit levels, and mean corpuscular hemoglobin^31^. Additionally, they contribute to higher whole-body protein and fat content^31^.

Moreover, organic acids play a significant role in protecting intestinal health and maintaining the intestinal barrier^32,33^. For example, dietary citric acid has been found to enhance phosphorus and calcium bioavailability and improve intestinal health in Nile tilapia^34^ and and juvenile turbot (*Scophthalmus maximus* L.)^35^. Similarly, fumaric acid supplementation promotes growth, improves intestinal villi morphometry, and reduces the prevalence of harmful intestinal bacteria in juvenile Nile tilapia^36^.

Given the promising effects of natural clays as feed additives, the present study aims to explore the impact of a natural clay product (Biocide) containing a combination of active components such as amino acids, organic acids, and silicon tetrahedron, on the growth performance and immune function of Nile tilapia.

## Materials and methods

### Analysis of Biocide

The amino acid and organic acid composition of Biocide were analyzed. Amino acid composition was analyzed using High-Performance Liquid Chromatography with Ultraviolet detection (HPLC-UV)^37,38^.

### Diet composition and preparation

The basal diet composition shown in Table 1 was the feed was formulated to meet the known nutritional requirements of the Nile tilapia NRC^39^. The experimental diets were created using the same basal formulation but with varying levels of Biocide: 0.0, 0.25, 0.5, 1, and 2 g kg^−1^). Biocide (EL-HODA Mining & Agricultural Development) is provided by Dr. Youssry Mohamed Ibraheem, Al Ahram Mining Company, Cairo, Egypt. The components of the experimental diets were mixed with water at a rate of 100 mL/kg diet. This mixture was then processed into a paste using a meat grinder and pelleted to create 2.0-mm pellets. The diets were subsequently dried at 57 °C for 24 h, packed in plastic bags, and stored at 4 °C.

**Table 1:** Ingredients and analyzed composition of the experimental diets (air-dry basis, g kg^–^^1^).

Ingredients	Biocide (g kg^–1^)				
	0.0	0.25	0.5	1.0	2.0
Fish meal (72%)	10.4	10.4	10.4	10.4	10.4
Soybean meal (44%)	42.98	42.98	42.98	42.98	42.98
Ground corn	20.32	20.32	20.32	20.32	20.32
Wheat bran	15.49	15.49	15.49	15.49	15.49
Cod fish oil	2.31	2.31	2.31	2.31	2.31
Vegetable oil	1.5	1.5	1.5	1.5	1.5
Vitamins and Mineral premix^1^	2.0	2.0	2.0	2.0	2.0
Di-Calcium phosphate	1.0	1.0	1.0	1.0	1.0
Starch	4.0	3.75	3.5	3.0	2.0
Biocide	0	0.25	0.5	1.0	2.0
Total	100	100	100	100	100
Chemical analysis (%)					
Dry matter	91.17	90.95	90.73	90.29	90.84
Crude protein	30.94	30.46	30.42	30.37	30.33
Crude fat	7.47	7.49	7.32	7.24	7.16
Ash	6.64	6.63	6.61	6.60	6.58
Fiber	4.89	4.87	4.87	4.82	4.79
NFE^2^	50.06	50.55	50.78	50.97	51.14
GE (MJ/kg)^3^	18.85	18.83	18.79	18.78	18.77

^1^Vitamin and mineral mixture each 1 kg of mixture contains: 4800 IU Vitamin A, 2400 IU cholecalciferol (Vitamin D), 40 g Vitamin E, 8 g Vitamin K, 4.0 g Vitamin B12, 4.0 g Vitamin B2, 6 g Vitamin B6, 4.0 g pantothenic acid, 8.0 g nicotinic acid, 400 mg folic acid, 20 mg biotin, 200 gm choline, 4 g copper, 0.4 g iodine, 12 g iron, 22 g manganese, 22 g zinc, 0.04 g selenium, 1.2 mg folic acid; 12 mg niacin; 26 mg D-calcium pantothenate; 6 mg pyridoxine HCl; 7.2 mg riboflavin; 1.2 mg thiamine HCl; 3077 mg sodium chloride (NaCl, 39% Na, 61% Cl); 65 mg ferrous sulphate (FeSO4. 7H2O, 20% Fe); 89 mg manganese sulphate (MnSO4, 36% Mn); 150 mg zinc sulphate (ZnSO4. 7H2O, 40% Zn); 28 mg copper sulphate (Cu- SO4 5H2O, 25% Cu); 11 mg potassium iodide (KI, 24% K, 76% I); 1000 mg Celite AW521 (acid-washed diatomaceous earth silica). w% on dry matter (DM) basis.

^2^Nitrogen-Free Extract (calculated by difference) = 100 – (protein + lipid + ash + fiber).

^3^Gross energy was calculated based on NRC (2011) as follows: protein, 23.6 MJ/kg; lipid, 39.4 MJ/kg; carbohydrates, 17.2 MJ/kg.

### Experimental culture conditions

A total of 300 healthy Nile tilapia, averaging 3.55 ± 0.01 g in weight, were sourced from the Fish Hatchery of the Central Laboratory for Aquaculture Research in Abbassa, Egypt. These fish were then allocated into 15 fiber tanks, each with a capacity of 150 L, and supplied with dechlorinated water. The fish were left for a two-week acclimation period and provided with a control ration (0.0 g kg^–1^ of Biocide) until the start of the trial. The feeding trial lasted for 90 days. Fish were weighed individually immediately prior to the start of the trial to obtain information about their initial weights and the initial tank biomass.

Continuous aeration was ensured in the tanks using an air stone connected to a central air compressor. Each tank underwent regular cleaning with 30% water replacement. Water quality parameters were monitored throughout the experiment to maintain optimal conditions for the fish. These parameters included water temperature (maintained between 25 and 27 °C), dissolved oxygen levels (maintained between 4.7 and 5.9 mg L^–1^), pH levels (maintained between 7.3 and 7.8), and total ammonia levels (maintained between 0.5 and 1.41 mg L^–1^).

### Growth performance and feed utilization

The growth performance and feeding rate of the fish were assessed by weighing them biweekly. Fish were fed three times daily, six days per week^40^. Initially, the feeding regimen was set at 5% of the fish’s body weight and gradually decreased to 3% over the last two weeks, with the feed amount adjusted weekly based on the fish’s weight^41^. Daily feed intake (FI) was calculated by subtracting the uneaten feed in each aquarium—collected 20 min after feeding, dried, and weighed—from the amount initially offered. On the 90th day, at the end of the experiment, the final weight and length of the fish were recorded, along with their initial weight measured on the first day of the experiment. Additionally, survival rates were recorded. The following equations were used to calculate growth performance^42,43^:

$${\text{Weight gain }}\left( {\text{g}} \right){\text{ }} = {\text{ W1 }}{-}{\text{ W}}0,{\text{ where W1 }} = {\text{ final body weight }}\left( {\text{g}} \right){\text{ and W}}0{\text{ }} = {\text{ initial body weight }}\left( {\text{g}} \right)$$

$${\text{Specific growth rate }}\left( {{\text{SGR}}\% /{\text{day}}} \right){\text{ }} = {\text{ }}\left( {\left( {{\text{Ln W1 }} - {\text{ Ln W}}0} \right)/{\text{ T}}} \right){\text{ }} \times {\text{ 1}}00,{\text{ where Ln }} = {\text{ natural logarithm and T }} = {\text{ time }}\left( {{\text{days}}} \right)$$

$${\text{Survival rate }}\left( \% \right){\text{ }} = {\text{ 1}}00{\text{ }} \times {\text{ }}\left( {{\text{fish no}}.{\text{ at the end }} \div {\text{ fish no}}.{\text{ stocked at the beginning}}} \right)$$

$${\text{FI }} = {\text{ Total FI per tank }} \div {\text{ number of fish}}$$

$${\text{FCR }} = {\text{ FI }}\left( {\text{g}} \right){\text{ }} \div {\text{ body weight gain }}\left( {\text{g}} \right)$$

$${\text{PER }} = {\text{ weight gain }}\left( {\text{g}} \right){\text{ }} \div {\text{ total protein intake }}\left( {\text{g}} \right)$$

$${\text{Apparent protein utilization }}\left( {{\text{APU}},{\text{ }}\% } \right){\text{ }} = {\text{ 1}}00{\text{ }} \times {\text{ }}\left( {{\text{protein gain in fish }}\left( {\text{g}} \right){\text{ }} \div {\text{ protein intake in diet }}\left( {\text{g}} \right)} \right)$$

$${\text{Energy utilization }}\left( {{\text{EU}},{\text{ }}\% } \right){\text{ }} = {\text{ 1}}00{\text{ }} \times {\text{ }}\left[ {{\text{gross energy gain }}\left( {\text{g}} \right){\text{ }} \div {\text{ gross energy intake }}\left( {\text{g}} \right)} \right]$$

### Diets and whole-body chemical composition

The approximate chemical composition of the experimental feeds, as well as the whole-fish bodies at the start and end of the experiment, was determined following AOAC^44^ guidelines. he initial body composition of 15 fish, separate from the original study population and stored at − 20 ºC, was analyzed. Three fish from each replicate were sacrificed for proximate body composition analysis.

Moisture content was measured by heating the samples at 105 °C until they reached a constant weight. Nitrogen content was determined using the Kjeldahl method, and crude protein content was estimated by multiplying the nitrogen percentage by 6.25. Total lipid content was measured through petroleum ether extraction using a Soxhlet apparatus for 16 h. Ash content was determined by incinerating the samples at 550 °C for 16 h in a muffle furnace. Gross energy content was calculated based on the parameters specified by NRC^39^.

### Blood and tissue sampling

At the end of the experiment (90 days), the fish underwent a 24-hour fasting period before sampling, followed by euthanasia using buffered tricaine methanesulfonate (MS-222) at a concentration of 250 mg L^–1^, followed by decapitation^45^. Whole blood samples were collected from the caudal peduncle using heparin (5000 IU mL^− 1^) from five fish per tank (15 fish per dietary treatment). Serum samples were collected using syringes without anticoagulant from a separate batch of fish, following the same sampling procedures. The serum was separated by centrifuging the clotted blood at 1267 x g for 15 min at 4 °C. The recovered serum samples were stored at − 20 °C until further analysis.

For oxidative stress biomarkers, liver and intestine samples were collected from a separate group of fish (five fish per replicate, 15 fish per group)^12,46^. The tissues were immediately transferred to cold phosphate-buffered saline and then stored at − 20 °C until use. The remaining parts of the proximal intestines from the same fish were used for measuring digestive enzymes; these parts were transferred into phosphate-buffered saline and kept at − 20 °C. Other portions of liver tissue, stomach, as well as the proximal and distal intestines, were placed in buffered formalin for histopathology^47^.

### Health performance parameters analysis

#### Blood profile and chemistry

After blood sampling, the erythrocyte count was determined using the Hayem solution at a ratio of 1:200 and a hemocytometer, following the method of Svobodová, et al.^48^. Hemoglobin concentration (Hb; g dL^–1^) was measured photometrically using the cyanohaemoglobin method. Packed cell volume (PCV %) was determined by centrifuging the blood at 10,000 xg for 5 min using a micro-capillary reader. Mean corpuscular hemoglobin volume (MCV; fL), mean cell hemoglobin (MCH; pg), and mean corpuscular hemoglobin concentration (MCHC; g dL^–1^) were calculated using the values obtained from the hematocrit and erythrocyte count. The differential leukocyte count, total leukocytes, and thrombocytes were determined from 2000 cells of a Giemsa-stained thin-layer blood smear.

Calorimetric determination of serum protein and serum albumin was performed using kits provided by El-Nasr Pharmaceutical Chemicals in Cairo, Egypt. Globulin values were roughly calculated by subtracting total protein values from albumin values. Glucose levels were determined using the enzymatic colorimetric method at 510 nm with commercial kits from Biodiagnostic in Egypt. Serum cholesterol, triglycerides, high-density lipoprotein (HDL), low-density lipoprotein (LDL), and very-low-density lipoprotein (VLDL) were measured using the Cholesterol Assay Kit - HDL and LDL/VLDL (ab65390) from Abcam, UK. Serum levels of urea were determined using urea assay kits from Sigma Aldrich, USA, and creatinine was measured using assay kits from Abcam, UK, spectrophotometrically at an absorbance of 570 nm.

#### Liver function test

The activities of AST, ALT, and ALP were measured at a wavelength of 540 nm using commercial kits (Biodiagnostic, Egypt).

#### Serum immune parameters

The concentration of serum nitric oxide was determined using a total nitrous oxide assay kit and the Griess reaction, as described by Sun, et al.^49^. Serum IgM antibody titers were measured using (ELISA kits, Cusabio Biotech Company, Wuhan, PRC). Lysozyme activity was determined according to the method outlined by Ellis^50^.

#### Oxidative stress biomarkers in liver and intestine

The total antioxidant capacity (TAC), catalase, and glutathione peroxidase (GPx) levels were measured in liver and intestinal homogenates at optical densities of 505 nm, 560 nm, and 340 nm, respectively, using Biodiagnostic kits from Egypt. MDA and lipid peroxidation markers were also measured in the liver and intestine tissues at an optical density of 534 nm using commercial kits from Biodiagnostic, Egypt.

### Digestive enzyme activities in the intestine

Homogenized tissues from the proximal intestine were processed in cold phosphate-buffered saline, followed by centrifugation at 5,000 g for 20 min at 4 °C. The resulting supernatants were utilized to assess lipase and amylase activities. Lipase activity was determined through commercially available kits (Abcam, UK) based on the hydrolysis of triglyceride substrates, leading to the formation of glycerol. Glycerol was quantified enzymatically by measuring the change in absorbance at an optical density of 570 nm^51^. Amylase activity, on the other hand, was measured at an optical density of 405 nm using commercially available kits from Abcam, UK.

### Histological examination

The liver, stomach, and intestines collected from five fish per replicate (15 fish per group) were promptly fixed in 10% neutral buffered formalin (pH = 7) for 24–48 h. Subsequently, the tissue samples were dehydrated and embedded in paraffin. Tissue sections, 5 μm in thickness, were cut using a microtome and stained with hematoxylin and eosin. Additionally, an alcian blue (pH 2.5) stain was employed to demonstrate mucous secretion in the stomach and intestine^52^. The alcian blue staining intensity was analyzed by counting the number of positively reacting mucous cells using ImageJ software.

### Statistical analysis

The normality of the data and homogeneity of variance were checked using the Shapiro–Wilk test and Levene test, respectively. One-way analysis of variance (ANOVA) was then employed to analyze the results. Post hoc tests were conducted using Duncan’s test in IBM SPSS version 27, with statistical significance set at *p*-values less than 0.05.

## Results

### Biocide analysis

The amino acid composition of Biocide revealed varying levels of amino acids, with glutamine, tyrosine, methionine, serine, and threonine ranging from 1,213 to 5,097 ppm (Supplementary Table 1). Among organic acids, fumaric acid and citric acid were the most abundant, with concentrations of 45,839 ppm and 14,390 ppm, respectively (Supplementary Table 2).

### Growth performance and feed utilization

Fish growth was significantly enhanced with Biocide supplementation compared to the control diet (Table 2). The highest final weight and weight gain percentage were achieved with a diet containing 1 g kg^–1^ of Biocide (Table 2). No significant differences in survival were observed among the treatments (Table 2).

**Table 2:** Growth performance and feed utilization of Nile tilapia fingerlings after feeding Biocide supplemented diets for 90 days.

Parameters	Biocide (g kg^–1^)				
	0.0	0.25	0.5	1.0	2.0
Initial weight (g)	3.58 ± 0.01	3.58 ± 0.01	3.57 ± 0.01	3.56 ± 0.01	3.55 ± 0.01
Final weight (g)	23.39 ± 0.43^d^	26.26 ± 0.74^c^	29.73 ± 0.47^b^	32.48 ± 0.67^a^	28.55 ± 0.48^b^
Weight gain (g)	19.81 ± 0.43^d^	22.68 ± 0.73^c^	26.16 ± 0.48^b^	28.92 ± 0.68^a^	25.00 ± 0.47^b^
SGR (%g / day)	2.23 ± 0.02^d^	2.37 ± 0.03^c^	2.52 ± 0.02^b^	2.62 ± 0.03^a^	2.48 ± 0.02^b^
Feed intake (g feed /fish)	28.48 ± 0.42^d^	29.93 ± 0.50^cd^	32.59 ± 0.90^b^	34.96 ± 0.82^a^	31.26 ± 0.67^bc^
FCR	1.43 ± 0.01^a^	1.32 ± 0.02^b^	1.24 ± 0.01^bc^	1.20 ± 0.02^c^	1.25 ± 0.04^bc^
FER	69.54 ± 0.77^c^	75.73 ± 1.18^b^	80.31 ± 0.92^ab^	82.74 ± 1.62^a^	80.08 ± 2.82^ab^
PER	2.44 ± 0.01^c^	2.68 ± 0.07^b^	2.83 ± 0.02^ab^	2.92 ± 0.08^a^	2.82 ± 0.08^ab^
Apparent protein utilization (%)	43.02 ± 0.58^b^	48.11 ± 0.46^a^	48.90 ± 0.97^a^	52.03 ± 2.36^a^	50.95 ± 1.88^a^
Energy utilization (%)	26.51 ± 0.78^b^	29.12 ± 0.10^ab^	29.53 ± 0.64^a^	31.17 ± 1.51^a^	30.27 ± 0.64^a^
Survival rate (%)	95.23	100	100	100	97.87

FCR, Feed conversion ratio; FER, Feed efficiency ratio; PER, Protein efficiency ratio; SGR, Specific growth rate. Results are expressed as mean ± SE. Different letters indicate significant differences at *p* less than 0.05.

FI increased significantly, and the FCR decreased significantly when fish were fed Biocide-supplemented diets (Table 2). It is noteworthy that the highest and lowest FCR values were obtained from the control and 1 g kg^–1^ Biocide diets, respectively. Furthermore, the feed efficiency ratio (FER) and PER values increased significantly in the Biocide-supplemented groups, with their highest values observed in the 1 g kg^–1^ Biocide diet (Table 2). There were no significant differences in APU and EU among all Biocide-supplemented diets.

### Whole-body composition

There were no significant differences in moisture, lipid contents, and total ash content in fish that received Biocide supplementation (Table 3). Crude protein contents increased significantly with increasing dietary Biocide levels, with the highest content observed in fish fed the 1 g kg^–1^ Biocide diet (Table 3).

**Table 3:** Whole body composition (%, on dry matter basis) of Nile tilapia fingerlings after feeding Biocide supplemented diets for 90 days.

Parameters	Biocide (g kg^–1^)				
	0.0	0.25	0.5	1.0	2.0
Moisture	72.76 ± 0.21	72.88 ± 0.85	73.80 ± 0.26	73.19 ± 0.29	73.01 ± 0.24
Crude protein	63.51 ± 0.27^b^	65.08 ± 0.91^ab^	65.16 ± 0.12^ab^	65.40 ± 0.41^a^	65.66 ± 0.53^a^
Total Lipids	21.44 ± 0.62	21.07 ± 0.81	20.93 ± 0.46	20.60 ± 0.69	20.27 ± 1.01
Ash	13.16 ± 0.26	13.44 ± 0.29	13.87 ± 0.86	14.03 ± 0.11	14.20 ± 0.05

Results are expressed as mean ± SE. Different letters indicate significant differences at *p* less than 0.05. Proximate analysis (%, on a dry matter basis) of the initial fish: moisture 74.38; protein 61.18; total lipid 18.73; and ash content 19.5.

### Health performance

#### Blood profile and chemistry

Supplementation of Biocide in tilapia diets led to a significant improvement (*p* < 0.05) in the hematological profile, specifically in terms of increased red blood cell (RBC) count, Hb, and PCV (Table 4). This improvement was most pronounced in the group fed a diet containing 1 g kg^–1^ of Biocide, followed by the group fed a diet with 2 g kg^–1^ of Biocide. Additionally, the percentage of lymphocytes was also influenced by the inclusion of Biocide in the diet, with a slight yet significant increase observed in the groups fed 1 and 2 g kg^–1^ of Biocide (Table 4).

**Table 4:** Hematological profile of juvenile tilapia fed diets supplemented with Biocide clay at various concentrations for 90 days.

Parameters	Biocide (g kg^–1^)				
	0.0	0.25	0.5	1.0	2.0
RBCs (10^12^L^–1^)	2.05 ± 0.22^b^	2.07 ± 0.02^b^	2.28 ± 0.16^ab^	2.73 ± 0.27^a^	2.56 ± 0.13^ab^
Hb (g dL^–1^)	6.40 ± 0.65^b^	6.10 ± 0.15^b^	7.30 ± 0.35^ab^	7.96 ± 0.80^a^	7.59 ± 0.40^ab^
PCV (%)	18.23 ± 1.88^b^	17.83 ± 0.63^b^	21.05 ± 0.99^ab^	22.93 ± 2.33^a^	21.9 0 ± 1.15^ab^
MCV (fl.)	74.93 ± 0.62	74.60 ± 1.53	75.50 ± 0.63	75.91 ± 0.32	75.53 ± 0.54
MCH (pg)	24.33 ± 0.21	25.00 ± 0.28	25.60 ± 0.23	25.90 ± 0.11	24.53 ± 0.20
MCHC (g dL^–1^)	29.66 ± 0.61	30.23 ± 0.23	29.86 ± 0.37	30.50 ± 0.49	30.46 ± 0.49
Platelets count (10^9^L^–1^)	811.0 ± 99.18	843.66 ± 76.23	845.66 ± 96.29	861.33 ± 101.26	883.0 ± 48.49
Leukogram WBCs (10^9^L^–1^)	33.00 ± 7.14^b^	37.40 ± 1.21^b^	33.93 ± 6.24^b^	68.16 ± 5.85^a^	68.93 ± 1.16^a^
Neutrophil (%)	42.66 ± 0.88	44.33 ± 0.33	41.33 ± 0.88	42.00 ± 1.15	42.00 ± 2.00
Lymphocytes (%)	42.66 ± 1.20^b^	42.66 ± 0.88^b^	43.00 ± 0.57^b^	49.66 ± 0.88^a^	51.00 ± 2.08^a^
Monocytes (%)	4.33 ± 0.33	5.00 ± 0.57	5.16 ± 0.32	4.83 ± 0.35	4.00 ± 0.57
Eosinophils (%)	2.35 ± 0.73	2.00 ± 0.00	2.33 ± 0.33	2.00 ± 0.00	2.40 ± 0.00
Basophils (%)	0.0 ± 0.0	0.0 ± 0.0	0.0 ± 0.0	0.0 ± 0.0	0.0 ± 0.0

RBCs, Red blood cells; Hb, Hemoglobin, PCV, Packed cell volume; MCV, Mean corpuscular volume; MCH, Mean cell hemoglobin; MCHC, Mean corpuscular hemoglobin concentration; WBCs, White blood cells. Results are expressed as mean ± SE. Different letters indicate significant differences at *p* less than 0.05.

Glucose concentration in the serum did not show a significant difference between the control and Biocide-supplemented groups (Table 5). Dietary Biocide for 90 days significantly affected serum lipid and lipoprotein levels in tilapia (*p* < 0.05; Table 5). Total cholesterol levels significantly decreased in all Biocide-supplemented groups (*p* < 0.05), with the lowest values recorded in the 1 g kg^–1^ Biocide group. Similarly, total triglyceride values showed a significant decrease (*p* < 0.05) in fish fed a Biocide-supplemented diet compared to the control. Biocide-supplemented fish exhibited significantly lower LDL and VLDL levels (*p* < 0.05) compared to the control (Table 5). On the other hand, fish fed a 1 g kg^–1^ Biocide-supplemented diet showed significantly higher (*p* < 0.05) HDL levels compared to the control and the other Biocide-fed group. Biocide-fed supplemented fish showed an increase in total protein, albumin, and globulin levels, but this increase was not significant (Table 5). Although urea and creatinine concentrations in the serum did not show a statistically significant difference compared to the control group, a non-significant (*p* > 0.05) decrease in their concentration was noticed in the Biocide groups (Table 5). The obtained results revealed a significant reduction (*p* < 0.05) in serum enzymes represented by ALT, AST, and ALP in Biocide-supplemented groups (Table 5).

**Table 5:** Serum biochemical profiling of juvenile tilapia fed diets supplemented with Biocide clay at various concentrations for 90 days.

Parameters	Biocide (g kg^–1^)				
	0.0	0.25	0.5	1.0	2.0
Glucose (mg dL^–1^)	178.21 ± 0.35^a^	174.70 ± 0.47^b^	170.81 ± 0.81^c^	178.25 ± 0.44^a^	173.18 ± 0.71^b^
Cholesterol (g dL^–1^)	220.56 ± 0.33^a^	189.72 ± 0.63^d^	214.82 ± 0.91^b^	148.22 ± 0.51^e^	200.32 ± 0.45^c^
Triglycerides (g dL^–1^)	142.70 ± 1.39^a^	89.52 ± 0.80^d^	93.77 ± 0.95^c^	81.24 ± 0.96^e^	104.61 ± 0.35^b^
HDL (mg dL^–1^)	42.85 ± 0.52^b^	44.31 ± 1.17^b^	43.05 ± 0.91^b^	48.55 ± 0.90^a^	37.21 ± 1.06^c^
LDL (mg dL–1)	153.68 ± 1.07^a^	148.01 ± 1.15^b^	147.92 ± 1.16^b^	145.95 ± 1.13^b^	135.80 ± 1.15^c^
VLDL (mg dL–1)	28.78 ± 1.11^a^	27.69 ± 1.40^a^	23.74 ± 1.08^b^	20.49 ± 0.94^b^	20.97 ± 0.92^b^
Total protein (g dL–1)	3.49 ± 0.42	2.78 ± 0.45	3.13 ± 0.80	3.95 ± 0.80	2.92 ± 0.74
Albumin (g dL–1)	1.36 ± 0.24	1.21 ± 0.26	1.39 ± 0.24	1.74 ± 0.43	1.34 ± 0.27
Globulin (g dL^–1^)	2.07 ± 0.57	2.53 ± 0.31	2.66 ± 0.65	2.88 ± 0.61	2.70 ± 0.17
Creatinine (mg dL^–1^)	0.39 ± 0.07	0.40 ± 0.11	0.37 ± 0.01	0.38 ± 0.01	0.37 ± 0.06
Urea (mg dL^–1^)	10.39 ± 0.99	9.32 ± 0.79	8.94 ± 0.53	8.12 ± 0.56	7.18 ± 0.54
ALT (U L^–1^)	28.76 ± 0.78^a^	25.77 ± 0.89^b^	24.17 ± 0.50^b^	25.31 ± 0.51^b^	24.72 ± 0.91^b^
AST (U L^–1^)	339.97 ± 0.43^a^	338.35 ± 0.45^a^	330.09 ± 0.53^b^	330.33 ± 0.45^b^	330.47 ± 0.60^b^
ALP (U L^–1^)	26.37 ± 0.54^a^	25.93 ± 0.87^a^	25.86 ± 0.65^a^	22.04 ± 0.66^c^	17.40 ± 0.47^d^
Nitric oxide (Umol L^–1^)	58.82 ± 0.57^d^	123.20 ± 1.03^c^	121.03 ± 0.61^c^	238.68 ± 0.83^a^	219.62 ± 0.45^b^
Lysozyme (U mg^–1^)	235.17 ± 0.55^e^	368.46 ± 0.95^c^	310.37 ± 0.86^d^	496.54 ± 0.51^a^	410.50 ± 0.35^b^
IgM (µg mL^–1^)	78.13 ± 0.65^e^	109.95 ± 0.50^d^	160.97 ± 0.66^c^	185.69 ± 0.86^a^	172.77 ± 0.96^b^

AST, aspartate aminotransferase; ALT, alanine aminotransferase; ALP, alkaline phosphatase; LDL, low-density lipoprotein; HDL, high-density lipoprotein; VLDL, very low-density lipoprotein. Results are expressed as mean ± SE. Different letters indicate significant differences at *p* less than 0.05.

#### Serum immunological parameters

The serum nitric oxide concentration significantly improved in all Biocide-fed groups compared to the non-fed control group (Table 5). A similar pattern of increase was observed in both lysozyme and IgM, with the highest values noticed in the 1 g kg^–1^ Biocide-supplemented fish (Table 5).

#### Oxidative stress biomarkers in liver and intestine

It was observed that including Biocide in the fish diet significantly increased the antioxidant-related enzymes (catalase and GPx) as well as TAC in both the liver and intestine of the 1 and 2 g kg^–1^ Biocide-supplemented groups (Table 6). On the other hand, the level of MDA in the liver and intestine significantly decreased in all Biocide groups, with notable reductions observed in the 1 and 2 g kg^–1^ Biocide-supplemented groups (Table 6).

**Table 6:** Oxidative enzyme activities in the liver and intestine of juvenile tilapia fed diets supplemented with Biocide clay at various concentrations for 90 days.

Parameters	Biocide (g kg^–1^)				
	0.0	0.25	0.5	1.0	2.0
Liver					
Catalase (U g^–1^)	8.29 ± 0.01^d^	8.90 ± 0.19^c^	11.94 ± 0.11^b^	11.96 ± 0.7^ab^	12.51 ± 0.32^a^
GPx mU (mU mL^–1^)	1.19 ± 0.04^c^	1.15 ± 0.05^c^	2.24 ± 1.64^b^	2.56 ± 0.13^a^	2.69 ± 0.04^a^
TAC (Mm L^–1^)	2.37 ± 0.01^d^	4.57 ± 0.60^c^	10.75 ± 0.24^ab^	10.84 ± 0.03^a^	10.45 ± 0.20^b^
MDA (nmol g^–1^)	23.63 ± 0.93^a^	22.61 ± 0.12^a^	19.85 ± 0.30^b^	12.65 ± 0.42^d^	17.12 ± 0.20^c^
Intestine					
Catalase (U g^–1^)	2.61 ± 0.06^d^	5.65 ± 0.25^c^	6.95 ± 0.25^b^	11.10 ± 0.11^a^	11.51 ± 0.39^a^
GPx (mU mL^–1^)	2.83 ± 0.02^c^	2.81 ± 0.28^c^	3.67 ± 0.10^b^	4.36 ± 0.05^a^	4.46 ± 0.29^a^
TAC (Mm L^–1^)	3.49 ± 0.25^d^	6.15 ± 0.16^c^	7.80 ± 0.28^b^	10.76 ± 0.04^a^	10.76 ± 0.24^a^
MDA (nmol g^–1^)	25.42 ± 0.37^e^	18.27 ± 0.42^c^	18.00 ± 1.17^c^	13.11 ± 0.35^a^	13.91 ± 0.58^a^

GPx, Glutathione peroxidase; MDA, Malondialdehyde; TAC, Total antioxidant capacity. Results are expressed as mean ± SE. Different letters indicate significant differences at *p* less than 0.05.

#### Digestive enzyme activities in the intestine

The activity of intestinal amylase, lipase, and protease enzymes was markedly and significantly enhanced (*p* < 0.05) in Biocide-supplemented groups, with the 1 g kg^–^^1^ Biocide group showing the best indication (Table 7).

**Table 7:** Digestive enzymes (amylase, protease, and lipase) activities in the intestine of juvenile tilapia fed diets supplemented with Biocide clay at various concentrations for 90 days.

Parameters	Biocide (g kg^–1^)				
	0.0	0.25	0.5	1.0	2.0
Amylase (U L^–1^)	596.66 ± 20.80^c^	564.66 ± 25.77^c^	530.66 ± 17.48^c^	812.65 ± 21.71^a^	835.56 ± 6.84^a^
Protease (ng mg^–1^)	5.65 ± 0.43^c^	8.81 ± 0.74^b^	10.59 ± 0.83^b^	19.92 ± 0.62^a^	19.88 ± 1.38^a^
Lipase (U L^–1^)	271.32 ± 7.41^c^	332.33 ± 30.57^bc^	351.34 ± 16.30^a^	358.65 ± 19.21^a^	351.10 ± 21.51^a^

Results are expressed as mean ± SE. Different letters indicate significant differences at *p* less than 0.05.

#### Histological findings

**Fig. 1:**
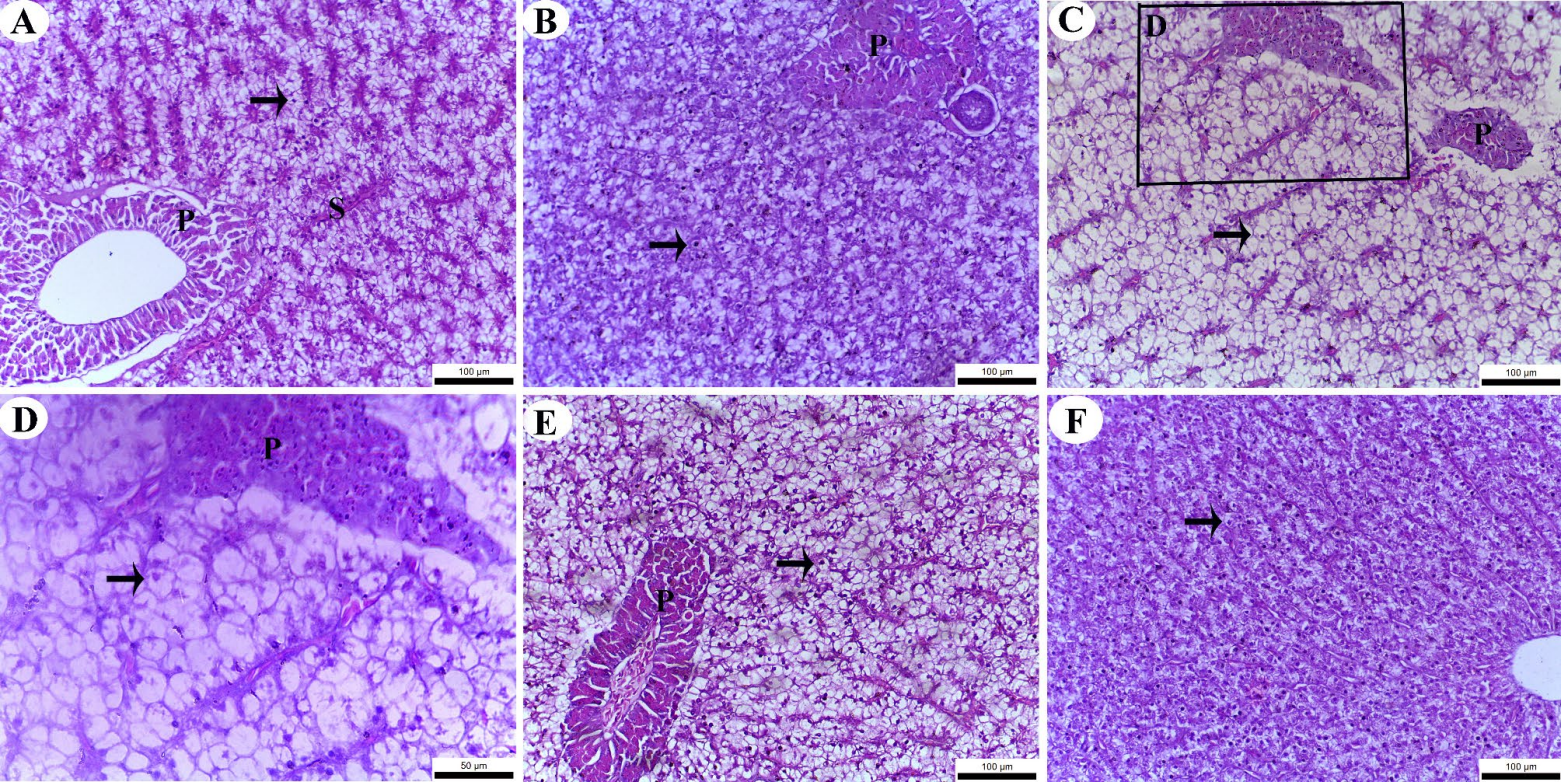
Liver parenchyma of Nile tilapia (*Oreochromis niloticus*) after being fed with various concentrations of Biocide for 90 days: **A** 0.25 g kg^–1^, **B** 0.5 g kg^–1^, **C** 1 g kg^–^^1^, **D** a magnified section from (C; 1 g kg^–1^), **E** 2 g kg^–1^, **F** 0 g kg^–^^1^ (control). The image shows hepatocytes (indicated by arrows), blood sinusoids (s), and the pancreas (P). The tissue is stained with H & E

The liver of fish exhibited a normal arrangement of hepatocytes, forming cords around the central vein, with blood sinusoids located between these cords. Hepatocytes were polygonal cells containing a large spherical nucleus and acidophilic cytoplasm (Fig. 1). Interestingly, lipid deposition within the hepatocyte cytoplasm increased following the feeding of diets supplemented with various Biocide clay formulations. This increase was correlated with a decrease in cytoplasmic acidophilic reaction, a phenomenon that became more pronounced with higher levels of Biocide treatment. This decrease in acidophilic reaction corresponds to the dissolution of lipids during slide staining. The highest lipid deposition (indicative of the lowest acidophilic reaction in the cytoplasm) was observed in fish supplemented with 1 g kg^–1^ of Biocide (Fig. 1C and D), whereas the control group showed the lowest lipid deposition in hepatocytes (Fig. 1F).

**Fig. 2:**
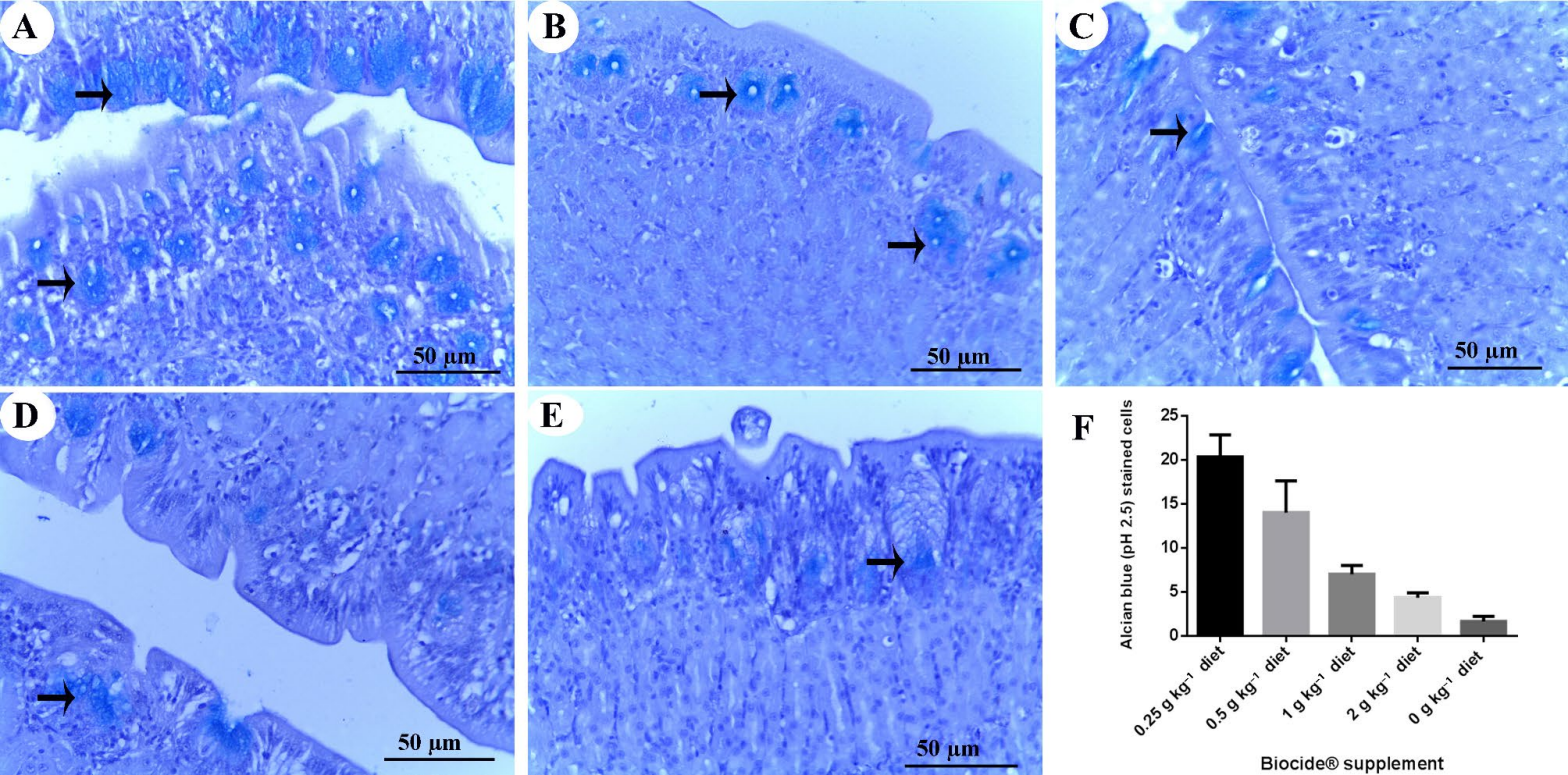
Stomach of Nile tilapia (*Oreochromis niloticus*) after being fed with various concentrations of Biocide for 90 days: **A** 0.25 g kg^–1^, **B** 0.5 g kg^–1^, **C** 1 g kg^–1^, **D** 2 g kg^–1^, **E** 0 g kg^–1^ (control). The image depicts mucous secretions within the gastric glands, as indicated by the arrows. The tissue is stained with alcian blue (pH 2.5). **F** A graph displays the difference in intensity (according to the counting of positively stained cells) of alcian blue (pH 2.5) staining in the gastric glands, with a *p*-value of 0.0244, presented as Means ± SD.

Furthermore, mucous secretion from gastric glands increased significantly after feeding diets supplemented with 0.25 g kg^–1^ of Biocide (Fig. 2A and D). However, this increase tended to decrease proportionally with increasing Biocide levels, ultimately reaching the lowest mucous secretion in the control group (Fig. 2B–F).

**Fig. 3:**
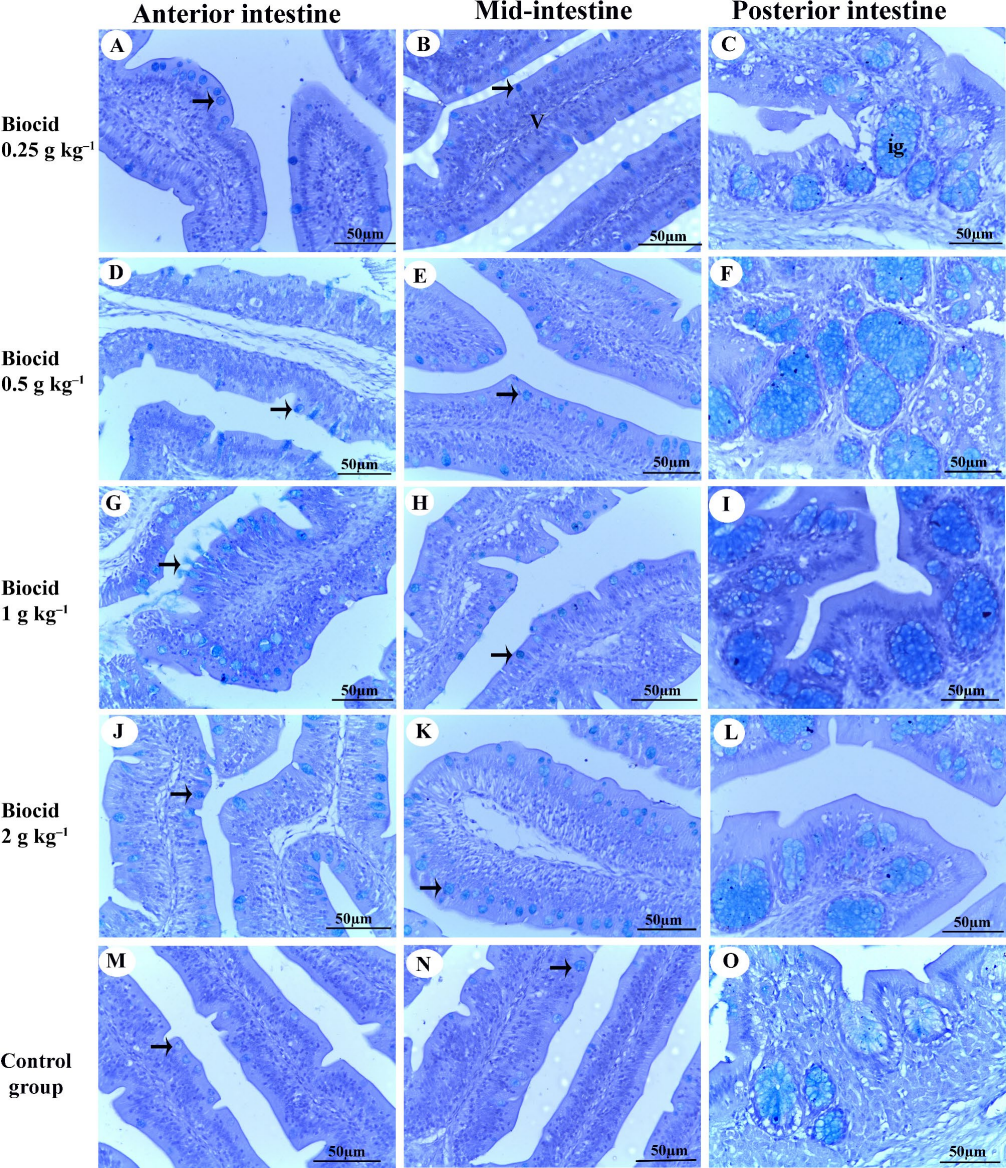
Intestine of Nile tilapia (*Oreochromis niloticus*) after being fed with various concentrations of Biocide for 90 days: **A**–**C** 0.25 g kg^–1^^1^, **D**–**F** 0.5 g kg^–1^, **G**–**I** 1 g kg^–1^, **J**–**L** 2 g kg^–^^1^, **M**–**O** 0 g kg^–1^ (control). The image shows goblet cells (indicated by arrows), villi (V), and the intestinal glands (ig). The tissue is stained with alcian blue (pH 2.5), and the scale bar represents 100 μm

In the intestinal villi of the anterior and mid-intestine, as well as in the intestinal glands of the posterior intestine, goblet cells displayed positive staining with alcian blue (pH 2.5) due to increased mucous secretion (Fig. 3). The mucous secretion from both goblet cells and intestinal glands reached high levels after feeding diets containing 1 g kg^–1^ of Biocide (Fig. 3G–I) compared to the control group, which showed the lowest mucous secretion from these structures (Fig. 3M–O).

The gastric mucosa and muscularis thickness of the stomach, along with the number of mucosal folds in the intestine, villous height and width of the intestine, thickness of intestinal muscles, and the number of mucous-secreting cells in the intestine—all these parameters, whether in the anterior, middle, or posterior intestine, exhibited the highest values in the fish fed 1 g kg^–1^ of Biocide and more modest values in those fed 2 g kg^–^^1^ of Biocide (Table 8).

**Table 8:** Histomorphology profile of the stomach and intestine of juvenile tilapia after receiving diets supplemented with Biocide clay at various concentrations for 90 days.

Parameters	Biocide (g kg^–1^)				
	0.0	0.25	0.5	1.0	2.0
Gastric mucosa and muscularis thickness of stomach (mm)					
Gastric mucosa	1.92 ± 0.13^b^	2.48 ± 0.17^ab^	2.60 ± 0.25^ab^	3.05 ± 0.11^a^	3.08 ± 0.38^a^
Gastric muscles	1.15 ± 0.09^b^	1.18 ± 0.10^b^	1.19 ± 0.03^b^	3.18 ± 0.24^a^	1.55 ± 0.10^b^
Number of mucosal folds of intestine^1^					
Anterior intestine	4.66 ± 0.33^d^	5.33 ± 0.33^bc^	6.33 ± 0.33^ab^	7.33 ± 0.33^a^	4.66 ± 0.33^d^
Mid- intestine	5.00 ± 0.57^ab^	4.00 ± 0.57^b^	6.00 ± 0.57^a^	6.66 ± 0.66^a^	3.33 ± 0.33^b^
Posterior intestine	2.33 ± 0.33^b^	2.66 ± 0.33^ab^	3.66 ± 0.33^ab^	4.00 ± 0.57^a^	4.00 ± 0.57^a^
Villous height of intestine (mm)					
Anterior intestine	3.00 ± 0.17^c^	3.15 ± 0.38^c^	4.52 ± 0.13^b^	7.90 ± 0.21^ba^	7.40 ± 0.22^a^
Mid- intestine	4.97 ± 0.65^b^	5.32 ± 0.14^b^	5.30 ± 0.16^b^	6.47 ± 0.26^a^	5.70 ± 0.25^ab^
Posterior intestine	2.45 ± 0.15^c^	2.50 ± 0.05^c^	2.90 ± 0.10^bc^	3.47 ± 0.20^a^	3.02 ± 1.18^ab^
Villous width of intestine (mm)					
Anterior intestine	1.02 ± 0.07^b^	1.29 ± 0.22^ab^	0.93 ± 0.12^b^	1.65 ± 0.10^a^	1.31 ± 0.09^ab^
Mid- intestine	0.97 ± 0.08^c^	1.47 ± 0.14^a^	1.55 ± 0.15^a^	1.38 ± 0.24^ab^	1.22 ± 0.05^ab^
Posterior intestine	1.67 ± 0.30^b^	2.45 ± 0.19^b^	2.82 ± 0.19^b^	3.72 ± 0.68^ab^	6.02 ± 1.54^a^
Thickness of intestinal muscles (mm)					
Anterior intestine	0.54 ± 0.05^c^	0.80 ± 0.04^b^	1.35 ± 0.06^a^	1.40 ± 0.14^a^	0.85 ± 0.02^b^
Mid- intestine	0.46 ± 0.03^c^	0.44 ± 0.02^c^	1.30 ± 0.04^a^	1.35 ± 0.06^a^	0.76 ± 0.07^b^
Posterior intestine	2.12 ± 0.37^a^	1.37 ± 0.08^b^	1.33 ± 0.02^b^	2.45 ± 0.06^a^	2.17 ± 0.04^a^
Number of mucous secreting cells^1^					
Anterior intestine	24.0 ± 2.08^c^	62.00 ± 0.57^b^	70.33 ± 0.33^b^	83.00 ± 2.30^a^	75.66 ± 2.96^a^
Mid- intestine	28.66 ± 1.85^c^	64.66 ± 0.88^b^	65.00 ± 1.15^b^	91.33 ± 3.38^a^	86.00 ± 1.00^a^
Posterior intestine	17.00 ± 4.58^c^	28.00 ± 0.57^b^	30.66 ± 1.20^b^	47.33 ± 2.18^a^	42.33 ± 1.45^a^

^1^Cell number per microscopic area in captured images at 20×. Results are expressed as mean ± SE. Different letters indicate significant differences at *p* less than 0.05. The data were obtained from tissues stained with H&E.

## Discussion

The findings revealed a significantly positive impact of Biocide, a natural clay containing silicon tetrahedrons and organic acids, on physiological performance in terms of growth and health. These improvements can be attributed to several factors inherent in natural clays. The act of consuming natural clays is a documented behavior in animals, known to benefit their health and performance by increasing the pH of the digestive tract, aiding in alleviating digestive disturbances, detoxifying harmful or unpalatable compounds in the feed, and supplying the body with minerals^53,54^. Natural compounds, such as clays, can directly or indirectly influence dietary components and the gut microenvironment, leading to improved growth performance and enhanced immune health.

It has been found that natural clays increase the digesta viscosity^55,56^. Increasing viscosity reduces the rate of digesta passage and increases intestinal fermentation in the gut^57^, could allow more digestion and nutrient absorption. The natural clay azomite has been shown to significantly enhance intestinal protease activity, consequently increasing protein retention and the overall crude protein content in largemouth bass (*Micropterus salmoides*)^58^. Similarly, a combination of sodium bentonite and oxy-cyclodextrin improved amylase, protease, and lipase enzyme activities, enhancing the growth of Nile tilapia^59^. Additionally, dietary silica nanoparticles have been found to enhance protein digestibility and retention in Nile tilapia^60^. This effect has also been observed with Biocide natural clay, which showed a notable enhancement in the activity of intestinal amylase and protease enzymes. Additionally, body crude protein contents increased significantly with rising levels of dietary Biocide, with the highest content observed in fish fed the 1 g kg^–1^ Biocide diet. The enhanced effect of natural clay is likely due to the slower digesta transit time in the gut, providing enzymes with the appropriate pH and essential cofactors like minerals.

Furthermore, natural clays have demonstrated the potential to act as carriers for biomolecules such as amino acids, aiding in their protection, transport, and support within animal nutrition^61,62^. This property may contribute to the efficient protection and delivery of nutrients, particularly amino acids, to gut mucosa. Additionally, clays can transport enzymes, enhancing their activities and delivery^63^, thus supporting more effective digestion.

The pH and intestinal microbiota are modulative factors that affect feed digestion, nutrient absorption, and availability. Natural clay has a buffering function and increases the pH of fecal matter, promoting a gut microenvironment conducive to better digestion^64,65^. Dietary montmorillonite clay has been shown to improve gut health, cellular structure, and histological architecture of the hepatopancreas and intestine. It also maintains healthy stomach bacterial richness and α-diversity index in shrimps during normal and diseased conditions^66^, and in turbot (*Scophthalmus maximus*)^67^. Montmorillonite also enhances intestinal barrier function, as evidenced by increased expression of barrier-related genes such as fascicilin II and integrin, along with increased villus height and width. Additionally, it augments the relative abundance of intestinal probiotics (*Lactobacillus*, *Ruegeria*, *Bacteroidales S24–7* group, and *Faecalibacterium*) and *Alloprevotella*, while decreasing the relative abundance of *Escherichia-Shigella*^68^. These changes collectively indicate a healthier gut microenvironment. Furthermore, modulating the gut microenvironment and microbiota population diversity has a significant impact on nutrient metabolism and absorption, including lipids, which in turn affects LDL and HDL levels^69,70^. These findings, along with the results of the current study, collectively demonstrate that natural clays, including Biocide, can sustain enhanced gut function by increasing muscularis thickness in the stomach and intestine, boosting goblet cell abundance, augmenting the number of mucosal folds in the intestine, and improving villous height and width in the intestine. The increased intestinal villi length observed in fish fed Biocide may be attributed to its abundant fumaric acid content. Studies have shown that intestinal villi length increases with higher dietary levels of fumaric acid, as demonstrated in Nile tilapia juveniles^36^. Ultimately, these effects enhance digestion, feed utilization and nutrient metabolism, increase the intestinal surface area for absorption, and thereby improve growth performance.

From another perspective, Biocide is rich in organic acids such as fumaric acid and citric acid. Fumaric acid has been shown to positively affect weight gain, feed efficiency ratio, and protein efficiency ratio in Nile tilapia^36^. Supplementing diets with compound acidifiers, such as organic acids (e.g., fumaric acid, formic acid, succinic acid, and malic acid), has been found to improve growth performance in juvenile channel catfish (*Ictalurus punctatus*). This improvement was accompanied by enhanced SOD activity, reduced MDA levels in the serum, and increased immune activities of lysozyme and acid phosphatase^33^. Furthermore, these organic acids significantly upregulated the expression of growth-related genes, including *growth hormone*, *growth hormone receptor*, *insulin-like growth factors 1* and *2*, and *insulin-like growth factor-binding proteins 1*, *2*, and *3* in the liver^33^. These genes play essential roles in promoting growth and weight gain^71,72^. Collectively, these findings could explain the positive impact of Biocide on the growth of Nile tilapia.

Additionally, fumaric acid significantly reduced the presence of Gram-negative bacteria, with no detection of Enterobacteriaceae in Nile tilapia fed fumaric acid for 28 days^36^. This bacterial family includes many opportunistic pathogens, such as *Enterobacter spp.*, *Klebsiella spp.*, *Escherichia coli*, *Salmonella spp.*, *Proteus spp.*, *Citrobacter spp.*, and *Serratia marcescens*, which are known to cause diseases ^73^. Furthermore, supplementation with organic acids demonstrated upregulation of key intestinal immune markers, including *transforming growth factor beta*, *interleukin-10*, *caspase-3*, *caspase-7*, *caspase-9*, *occludin*, *zonula occludens-1* and *− 2*, *claudin-12*, *claudin-15a*, and *claudin-15b*^33^. This indicates a positive regulation of the immune response.

Citric acid is a potent modulator of fish growth and intestinal health^30,35,74^. Dietary citric acid significantly increases pepsin activity and improves phosphorus bioavailability in juvenile turbot^35^. It also enhances protease and amylase activities, improves intestinal microbiota composition, and increases intestinal villus height^75^. Furthermore, citric acid boosts growth performance in *Carassius auratus gibelio*^76^. It promotes the population of beneficial intestinal microorganisms and upregulates key genes that maintain the integrity of the intestinal tight junction barrier^32^. Citric acid also mitigates dysfunctions caused by soybean meal replacement in large yellow croaker (*Larimichthys crocea*), such as reduced alkaline phosphatase, leucine-aminopeptidase, and Na^+^, K^+^-ATPase activities, as well as declines in specific growth rate, feed efficiency, and phosphorus and protein retention. Additionally, it restores phosphorus and zinc concentrations in the whole body and intestines^77^.

Citric acid alleviates soybean meal-induced intestinal inflammation in turbot by exerting an anti-inflammatory effect on Toll-like receptor-mediated activation of NF-κB and interferon regulatory factor-3 signaling pathways^74^. It also enhances the health of intestinal tight junctions through the Toll-like receptor-mediated p38 and Jun N-terminal kinase pathways. Specifically, citric acid downregulates the expression of the pore-forming tight junction protein claudin-7 and pro-inflammatory cytokines (e.g., *tnf-α* and *ifn-γ*). Simultaneously, it upregulates the expression of the anti-inflammatory cytokine transforming growth factor-beta1 and tight junction proteins involved in reducing paracellular permeability, such as claudin-3, claudin-4, occludin, tricellulin, and zonula occludens-1^74^.

This protective effect is further supported by increased total antioxidative capacity and decreased MDA levels in the distal intestine. Citric acid also upregulates the expression of antioxidant and cellular repair genes, including *sod*, *gpx*, *heme oxygenase 1*, *proliferating cell nuclear antigen*, and mucins, while downregulating stress and apoptosis-related genes such as *p53*, *protein kinase C*, and *caspase-3*^78^. These changes help maintain an intact intestinal mucous layer^78^. This effect aligns with the observed impact of Biocide on gut mucous secretion in Nile tilapia. Dietary organic acids have been shown to increase the number of goblet cells in Nile tilapia^79^. The mucus layer, composed of water-insoluble glycoproteins secreted by goblet cells, plays a vital role in nutrient absorption, immune defense, and the prevention of bacterial adhesion. Additionally, this mucus contains various antimicrobial components, such as lectins, immunoglobulins, lysozymes, and other bioactive substances^80^.

Notably, combining citric acid with AZOMITE, a hydrated aluminosilicate mineral, significantly enhances growth performance, protein and lipid retention, antioxidant responses (e.g., GPx, SOD, catalase, and total antioxidant capacity), and lysozyme activity in largemouth bass (*Micropterus salmoides*)^75^. This combination also improves disease resistance against *Aeromonas hydrophila*^75^. Similar benefits have been observed in *Carassius auratus gibelio*^76^. Moreover, citric acid reduces the relative abundance of the pathogenic *Vibrio* genus in the intestine^78^.

On the other hand, Biocide, as a natural clay, may exert mechanical effects. For instance, dietary kaolin supplementation enhanced the counts of RBC and WBC, percentage of lymphocytes and eosinophils, serum levels of albumin and globulin, and immunoglobulin levels in *Ctenopharyngodon idellus*. It also increased the activities of lysozyme, complement, and SOD, improving resistance against *Aeromonas hydrophila* infection, while not significantly affecting phagocytic activity^81^. Concurrently, the natural clay Biocide, particularly at a dosage of 1 g kg^–1^, increased serum lysozyme and IgM, and antioxidant-related enzymes such as catalase and GPx, as well as TAC in both the liver and intestine, while decreasing MDA in these organs.

Natural clays have exhibited antimicrobial activities against both Gram-positive and Gram-negative bacteria, as well as *Candida* sp ^53,82–84^. These antimicrobial properties can vary among different types of clay due to chemical reactions^53,85^. Overall, it is evidence that natural clays possess physical and chemical properties that can enhance growth and immune responses.

Biocide has been found to naturally contain essential amino acids such as glutamine, tyrosine, methionine, serine, and threonine, which play vital roles in growth and immune response. Beyond their essential functions in growth, certain amino acids, even at low levels, can enhance immune function and overall health. For instance, threonine at low dietary levels has been shown to improve immune and antioxidant responses, as well as modulate growth in fish^86,87^. Similarly, glutamine, methionine, tyrosine, and serine play critical roles in supporting growth and immune response under normal conditions and during disease challenges. These amino acids, when included in dietary levels slightly above those required for growth, have demonstrated the ability to enhance innate immune and anti-inflammatory responses, reduce stress markers, and improve antioxidant activity^88–91^.

Based on our results, feeding Nile tilapia diets supplemented with Biocide increased lipid deposition in hepatocytes, as evidenced by the reduced acidophilia of their cytoplasm. Similarly, Abd El-Naby, et al.^92^ found that feeding Nile tilapia diets containing chia seeds powder also led to increased lipid deposition in hepatocytes. Additionally, Li, et al.^93^ observed that feeding loach (*Misgurnus anguillicaudatus*) diets with varying levels of soybean oil markedly increased lipid deposition in hepatocyte cytoplasm.

Natural clays are effective adsorbents that bind toxins, such as mycotoxins and heavy metal ions^65^. Clays and silicates have characteristics that allow them to bind minerals like calcium and other negatively charged minerals. This, in turn, can affect their bioavailability and absorption. However, these effects do not necessarily translate into negative impacts on the nutritive value of feed when included within it^65,94^. For example, research has shown that natural clay, such as calcium and sodium montmorillonite, does not significantly affect the dietary nutrient and mineral bioavailability, nor does it alter body mineral contents, even during normal physiological stress^95^. This suggests a certain selectivity of natural clays in binding harmful molecules related to health.

The adsorption function of natural clay reduces toxins that may be found in feed, leading to less stress and maintaining normal antioxidant defense. Nano-zeolite, on the other hand, reduces the damage induced by aflatoxin B1, thus helping to maintain low levels of serum ALT, AST, and ALP, as well as mitigating DNA damage and fragmentation^96^, even under normal conditions^67^. In Nile tilapia exposed to diazinon or cadmium, dietary bentonite clay exhibited a hepatorenal protective role with antioxidative properties. It maintained healthy levels of serum ALT, AST, ALP, urea, creatinine, catalase, SOD, reduced glutathione, and MDA^97,98^. Dietary organic acids have been shown to reduce serum levels of glutamic oxalacetic transaminase and glutamic pyruvic transaminase^33^. Given the organic acid content of Biocide and its association with favorable levels of ALT, AST, and ALP, it suggests that natural clays like Biocide may exhibit a hepatoprotective effect.

In addition, dietary combinations of organic acids increased total cholesterol and HDL levels in the serum while reducing triglyceride levels in juvenile channel catfish^33^. Similarly, zinc oxide supported on kaolinite improved the blood lipid profile in Nile tilapia^99^. Supplementation with Biocide reduced serum cholesterol and triglyceride levels while elevating HDL levels, indicating that Biocide, with its balanced formulation of organic acids, supports a healthy blood lipid profile and overall health.

## Conclusion

Natural clays have demonstrated significant potential in enhancing fish growth and overall health. The dietary natural clay Biocide, when used at different concentrations (0.25, 0.5, 1, and 2 g kg^–1^), notably improved growth performance and final weights, increased digestive enzyme activities, and elevated protein content in the entire body. Moreover, it enhanced antioxidant and immune responses at varying levels and promoted gut function. The most pronounced effects were observed at a concentration of 1 g kg^–^1.

## Electronic supplementary material

Below is the link to the electronic supplementary material.

Supplementary Material 1

## Data Availability

All relevant data are available from Amel M. El Asely (amel.alaasly@fvtm.bu.edu.eg) upon reasonable request.
