# Supplementary material for: Impact of dietary Biocide clay on growth, physiological status, and histological indicators of the liver and digestive tract in Nile tilapia (Oreochromis niloticus)
Source: Sci Rep. 2025 Feb 13;15:5311. doi: 10.1038/s41598-025-89042-9 (PMC11821997; doi:10.1038/s41598-025-89042-9)
Supplement: Supplementary file 1 — Supplementary Material 1 [file 41598_2025_89042_MOESM1_ESM.docx]

**Supplementary Table 1.** Aminon acid composition of Biocide

| Components | Amount (ppm) |
| --- | --- |
| Glutamine | 5097.88 |
| Tyrosine | 1599.89 |
| Methionine | 1496.87 |
| Serine | 1339.33 |
| Threonine | 1213.20 |
| Alanine | 797.77 |
| Iso leucine | 751.70 |
| Phenylalanine | 675.92 |
| Leucine | 647.35 |
| Aspartic | 609.18 |
| Histidine | 430.24 |
| Valine | 364.25 |
| Proline | 289.54 |
| Glycine | 217.83 |
| Cysteine | 214.57 |
| Arginine | 156.71 |
| Tryptophane | 25.57 |
| Cysteine | 8.34 |

**Supplementary Table 2.** Organic acid composition of Biocide

| Components | Amount (ppm) |
| --- | --- |
| Fumaric acid | 45839.73 |
| Citric acid | 14390.25 |
| Succinic acid | 6941.25 |
| Oxalic acid | 1251.37 |
| Maleic acid | 772.62 |
| Tartaric acid | 383.39 |
